# Evolutionary diversification of secondary mechanoreceptor cells in tunicata

**DOI:** 10.1186/1471-2148-13-112

**Published:** 2013-06-04

**Authors:** Francesca Rigon, Thomas Stach, Federico Caicci, Fabio Gasparini, Paolo Burighel, Lucia Manni

**Affiliations:** 1 Dipartimento di Biologia, Università degli Studi di Padova, via U. Bassi 58/B, I-35121, Padova, Italy; 2 Institut für Biologie, AG Vergleichende Zoologie, Humboldt-Universität zu Berlin, Philippstr. 13, Haus 2, D-10115, Berlin, Germany

**Keywords:** Cladistic analysis, *Oikopleura albicans*, *Oikopleura dioica*, Sensory cells

## Abstract

**Background:**

Hair cells are vertebrate secondary sensory cells located in the ear and in the lateral line organ. Until recently, these cells were considered to be mechanoreceptors exclusively found in vertebrates that evolved within this group. Evidence of secondary mechanoreceptors in some tunicates, the proposed sister group of vertebrates, has recently led to the hypothesis that vertebrate and tunicate secondary sensory cells share a common origin. Secondary sensory cells were described in detail in two tunicate groups, ascidians and thaliaceans, in which they constitute an oral sensory structure called the coronal organ. Among thaliaceans, the organ is absent in salps and it has been hypothesised that this condition is due to a different feeding system adopted by this group of animals. No information is available as to whether a comparable structure exists in the third group of tunicates, the appendicularians, although different sensory structures are known to be present in these animals.

**Results:**

We studied the detailed morphology of appendicularian oral mechanoreceptors. Using light and electron microscopy we could demonstrate that the mechanosensory organ called the circumoral ring is composed of secondary sensory cells. We described the ultrastructure of the circumoral organ in two appendicularian species, *Oikopleura dioica* and *Oikopleura albicans*, and thus taxonomically completed the data collection of tunicate secondary sensory cells. To understand the evolution of secondary sensory cells in tunicates, we performed a cladistic analysis using morphological data. We constructed a matrix consisting of 19 characters derived from detailed ultrastructural studies in 16 tunicate species and used a cephalochordate and three vertebrate species as outgroups.

**Conclusions:**

Our study clearly shows that the circumoral ring is the appendicularian homologue of the coronal organ of other tunicate taxa. The cladistic analysis enabled us to reconstruct the features of the putative ancestral hair cell in tunicates, represented by a simple monociliated cell. This cell successively differentiated into the current variety of oral mechanoreceptors in the various tunicate lineages. Finally, we demonstrated that the inferred evolutionary changes coincide with major transitions in the feeding strategies in each respective lineage.

## Background

Tunicates include sessile ascidians, planktonic appendicularians and thaliaceans and are considered a key group in the investigation of vertebrate evolution. Indeed, recent chordate phylogenies based on molecular data place tunicates as the sister group of vertebrates, with the cephalochordates at the most basal position [1,2]. During the last few years, several studies on ascidians and thaliaceans have been focused on the identification and description of tunicate sensory systems to understand the evolution of vertebrate sensory organs. The latter are particularly elaborate, and a better understanding of their structural origin and evolution will help researchers to comprehend their organisation and function.

Secondary sensory cells lack their own axon but form synapses with other neurons at the level of their basal plasmalemma. In ascidians, these cells have been hypothesised as possible homologues of vertebrate secondary sensory cells, namely the hair cells of the ear and the lateral line. In all ascidians analysed so far, sensory cells are located in the oral aperture and are arranged to form a structure called the coronal organ, which is composed of a row of cells bordering the oral tentacle surface [3]. Its role is to monitor inflowing water during the feeding process [4]. Coronal sensory cells show considerable variability in different species [3,5,6], especially regarding the apical cell surface, which can exhibit one or more cilia that may be surrounded by microvilli or stereovilli of similar or different lengths. In thaliaceans, the coronal organ was described in *Doliolum nationalis* and *Pyrosoma atlanticum*, which represent the orders Doliolida and Pyrosomatida [7]. The coronal organ is absent in the salp *Thalia democratica*, and it has been hypothesised that this condition is due to adoption of a different feeding system by this group of animals. Contrary to ascidians, doliolids, pyrosomes and salps actively pump a large amount of water into their mouths utilising muscular contractions instead of pharyngeal ciliary beating.

Secondary sensory cells have been identified also in the mouths of appendicularians. In *Oikopleura dioica*, they are present as ciliated cells [8] which constitute a ring of ciliated mechanoreceptor cells. The ring is oblique with respect to the transversal plane of the animal: caudally, it is located in the roof of the oral cavity, and ventrally, it reaches the tip of the lower lip. Unfortunately, there is no information at present on the detailed cytoarchitecture of these cells. There is little information about the organisation of the apical sensory structure of these cells, and we do not know whether they are monociliated or multiciliated, with or without microvilli or stereovilli. Circumoral ring cells are connected to the brain through the ventral branch of the second nerve [8]. It is also known that the cells located on the lower lip have the ability to detect mechanical stimuli and provoke a response of ciliary reversal in the spiracles, which are rings of ciliated cells responsible for water pumping [9]. Similarly to the coronal organ on the oral tentacles of other tunicates, the lip receptors in appendicularians prevent larger particles from entering the mouth via incurrent seawater flow. More recent authors have confirmed that these receptors respond to tactile stimuli [10,11]. In contrast to the remaining tunicates, appendicularians possess so-called Langerhans cells, which are other secondary mechanoreceptors located in the posterior of the trunk. When stimulated, Langerhans cells trigger the escape response of the animal [12].

To improve our understanding of the evolution of oral secondary sensory cells in chordates, we work towards two goals. First, we investigate the structural details of the circumoral ring cells in the appendicularian species *Oikopleura dioica* and *Oikopleura albicans*. From these we obtain the cytological data necessary to compare the circumoral ring cells to the sensory cells in the coronal organs of ascidians and thaliaceans and complete the picture of the oral secondary sensory cells in tunicates. Second, we trace the evolution of secondary sensory cells within tunicates by conceptualising a data matrix based on morphological characters of secondary sensory cells in tunicates. Representative species of both cephalochordates and vertebrates are used as outgroups.

Within chordates, cephalochordates possess several types of secondary sensory cells spread in different head regions [13]. In particular, the oral spines are sensory structures placed around the mouth; if stimulated, they are able to provoke a rejection response and the expulsion of water [14-16]. On the basis of their position and morphology, oral spines were proposed to be homologues to vertebrate taste buds. Moreover, a possible homology between the nerve plexus contacting these cells and the adoral nerves and ganglia of echinoderms [17] has also been hypothesised on the basis of shared features between them. It is noteworthy that echinoderm and hemichordate mouths do not display any secondary sensory cells [18]. Lacalli and co-authors thus proposed that amphioxus has retained some features typical of hemichordates and echinoderms, such as the plexus-like intraepidermal organisation of the nerve network, while also acquiring new structures in the form of oral spine secondary sensory cells as in other chordates.

Vertebrate hair cells of the ear and lateral line were believed to be exclusive for vertebrates and to have evolved within them because of their typical morphology and development [19]. They develop from a number of embryonic placodes [20,21]; these ectodermal thickenings are characterised by the expression of some common placodal genes (*i*.*e*., *Eya1*, *Six1*) and others more specifically related to the single type of placode [20,21]. In tunicates, embryonic territories marked by the same set of genes are able to give rise to sensory organs that were recognised in ascidians [22,23] and appendicularians [24]. The ascidian stomodeal placode is located anterior to the neural plate border and the oral siphon and coronal organ develop from it [25,26]. In addition to *Eya* and *Six1* genes, the ascidian stomodeal placode also expresses *Pitx*[27]. In vertebrates, *Pitx* characterises the extended anterior placodal area and the derivative placodes (adenohypophyseal, lens and olfactory placodes) [21] from which secondary sensory cells do not differentiate. Similarly to ascidians, the mouth in *O*. *dioica* derives from the stomodeal placode with a comparable gene expression pattern to that of ascidians [24,28]. In contrast to tunicates, true placodes have not been identified in cephalochordates; however, several studies demonstrated that some broad ectodermal regions are characterised by the expression of typical placodal genes and are able to differentiate into both primary neurons and secondary sensory cells [29]. The ability to differentiate neurons from the neural ectoderm has been suggested to have been present in all chordates; this would initially exist in a broader region, as observed in cephalochordates, and subsequently would be refined to restricted, specialised regions differentiating placodes, as found in tunicates and vertebrates [29,30].

Our data demonstrate that the circumoral ring of cells in appendicularians can be considered homologous to the coronal organs of ascidians and thaliaceans. These cells are located in a position corresponding to the coronal organs and are composed of secondary sensory cells possessing the same mechanoreceptor function. Moreover, our phylogenetic analysis shows that the chordate oral secondary sensory cells are derived from a simple monociliated prototype cell from which the current diversity of sensory cells progressively evolved.

## Methods

Specimens of *Oikopleura dioica* and *Oikopleura albicans* were collected in front of the Zoological Station in Ville franche-sur-Mer (France). In addition, developmental stages of *Oikopleura dioica* were obtained in the SARS High Technology Center in Bergen, Norway. Precisely timed stages were obtained by mixing ripe eggs and sperm and pipetting the animals directly into the primary fixative (1.7% glutaraldehyde buffered in 0.2 M sodium cacodylate buffer, pH 7.4, plus 1.7% NaCl) on ice.

### Transmission electron microscopy

Specimens of juveniles and adults were anesthetised with 0.02% MS222 at 4°C. After complete relaxation, specimens were fixed in the primary fixative or in 1% glutaraldehyde buffered in phosphate buffer (1.28 mM NaH_2_PO_4_ plus 5.38 mM Na_2_HPO_4_, pH 7.4). After post-fixation in 1% OsO_4_ in 0.2 M cacodylate buffer, specimens were dehydrated and embedded in Epon Araldite 812. Thick sections (1 μm) were counterstained with toluidine blue; thin sections (80 nm) were given contrast by staining with uranyl acetate and lead citrate. Micrographs were taken with a Hitachi H-600 (operating at 75 kV) and FEI Tecnai G^2^ electron microscope (operating at 100 kV). All photos were collected and labelled in Corel Draw X3.

### Scanning electron microscopy

Specimens were fixed in glutaraldehyde solution as described for transmission electron microscopy. After post-fixation and dehydration, they were critical-point dried, sputter-coated with gold, and observed under a Cambridge Stereoscan 260 and under a Fei Quantum 200 scanning electron microscopes. Micrographs were collected and then labelled in Corel Draw X3.

### Phylogenetic analysis

#### Construction of morphological character matrix

We constructed a matrix based on 19 characters derived from detailed ultrastructural studies of oral secondary sensory cells using Mac Clade 4.08 [31]. Phylogenetic analysis allows for the detection of phylogenetic information present in the examined structures to complement both morphological and molecular matrices. Character definitions and descriptions of character states are detailed in the Results section. Character coding was strictly binary to maximise information content [32].

#### Analyses

All phylogenetic analyses were performed using PAUP* (version 4.0b10) [33]. Parsimony analyses were conducted using the branch and bound option. A strict consensus tree and a 50% Majority Rule consensus tree were calculated. Jackknife values were calculated for 1000 replicates using a heuristic search strategy with n = 10 random addition sequence replicates, TBR branch swapping, retaining all optimal trees, and 30% random character deletion. We tracked transformations of character states in the resulting trees using Mac Clade 4.08 with standard settings including ACCTRAN optimisation [31].

## Results

### Secondary sensory cells in the mouth of *Oikopleura dioica* and *Oikopleura albicans*

**Figure 1:**
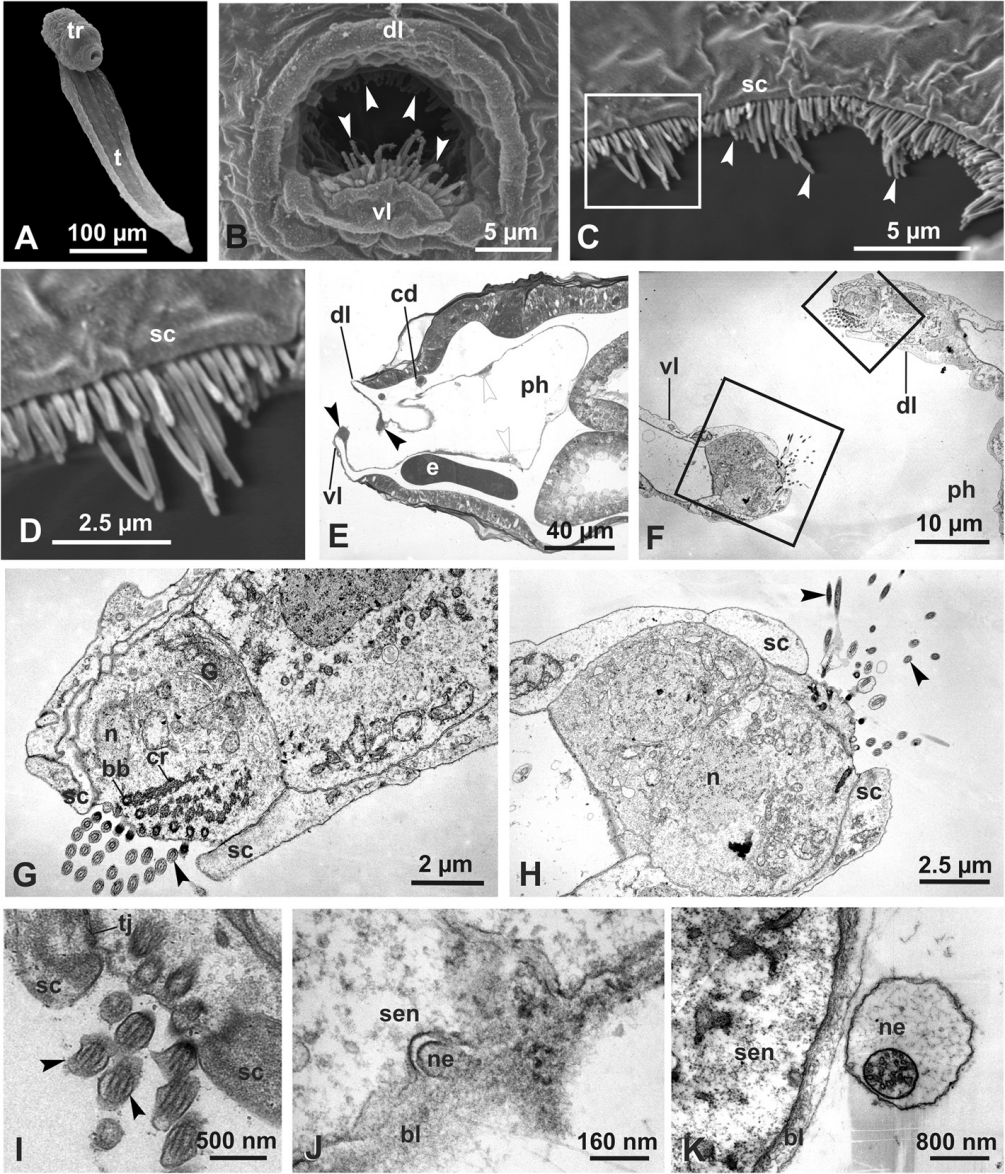
**Secondary sensory cells in *Oikopleura dioica.*** (**A**-**D**) Scanning electron micrographs. (**A**-**B**) Juvenile 26 hours old (**A**) and detail of the mouth (**B**) to show the cilia of sensory cells belonging to the circumoral ring. Arrowheads: cilia belonging to sensory cells; dl: dorsal lip; t: tail; tr: trunk; vl: ventral lip. (**C**-**D**) Mouth of an adult animal showing cilia (white arrowheads) of sensory cells located on the dorsal lip. Cilia are of different lengths; this confers a wavy arrangement to the circumoral ring. Note that the apical membrane of supporting cells (sc) forms a crest delimiting the sensory bundle. The square area in **C** is enlarged in **D** to show that each sensory cell possesses a number of ciliary rows that form the sensorial apparatus. (**E**) Sagittal section of the head showing the ventral (vl) and dorsal (dl) lips and the circumoral ring (black arrowheads); white arrowheads: perypharyngeal band; cd, ciliated duct of the neural gland; e, endostyle; ph, pharynx. (**F**-**K**) Transmission electron microscopy of circumoral organ. The organ is formed by a single cell row dorsally located on the roof of the oral cavity and ventrally on the tip of the ventral lip (vl). Squared areas in **F** are enlarged in **G** and **H** to show dorsal and ventral receptor cells, respectively. The hair bundle is multiciliated and delimited by apical extensions of supporting cells (sc). In I cilia show a conventional 9+2 microtubular arrangement. G shows a dense, short basal body (bb) with developed ciliary rootles (cr). Arrowheads: cilia of sensory cells; bl, basal lamina; dl, dorsal lip; G, Golgi complex; n, nucleus; ph, pharynx; tj, tight junction. Note that in J and K neurites (ne) are very close to the sensory cell membrane (sen).

**Figure 2:**
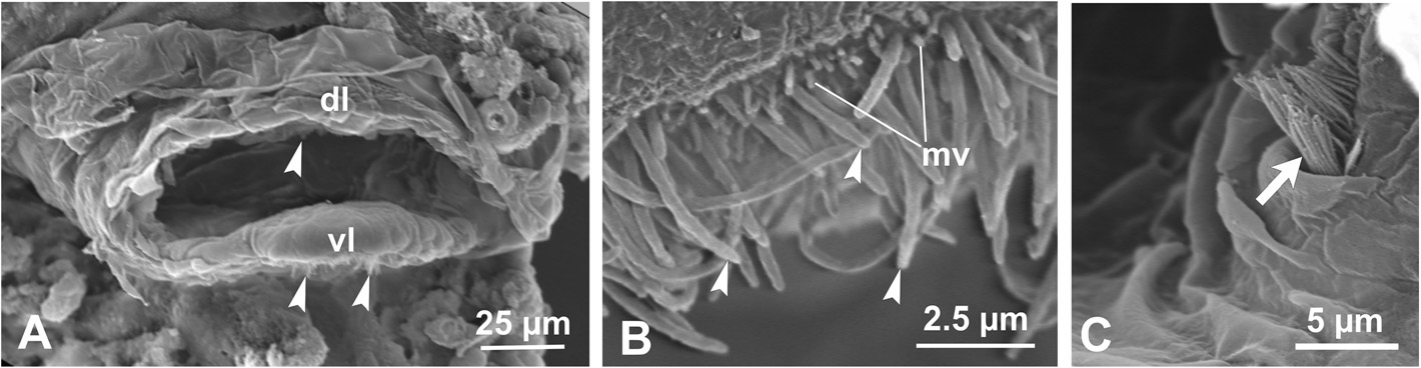
**Secondary sensory cells in *Oikopleura albicans.*** (**A**-**C**) Scanning electron microscopy. The mouth (**A**) is furnished with a dorsal (dl) and a ventral (vl) lip bearing the circumoral ring (arrowheads pointing to cilia). Cilia are of different lengths and, in the ventral lip (**B**), are accompanied by short microvilli (mv). In this species, the circumoral ring is not continuous, but interrupted at the lateral edges; in (**C**), left limit of the upper row of sensory cells (arrow).

In both *O*. *dioica* and *O*. *albicans*, the mouth is delimited by two dorsal and ventral lips, of which the ventral lip protrudes anteriorly. Sensory cells are arranged to form the circumoral ring; this structure is continuous in *O*. *dioica* while it is interrupted at its lateral corners in *O*. *albicans* (Figures 1–2). Scanning electron microscopy analysis reveals that in both species, sensory cells possess apical cilia arranged in multiple rows. The cilia are of different lengths. In each cell, the longest cilium is situated in the centre. The lateral cilia are gradually shorter, conferring a wavy outline to the circumoral ring (Figure 1C-D). In *O*. *albicans*, cilia of ventral lip receptors are accompanied by short microvilli (Figure 2B).

Transmission electron microscopy observations were performed in *O*. *dioica*. Sagittal sections of the mouth region reveal that the circumoral ring consists of a single row of sensory cells (Figure 1E-H). The sensory cells are flanked by non-ciliated supporting cells that appear C-shaped in cross sections. These supporting cells form a continuous groove that harbours the apical cilia of the sensory cells throughout the circumoral ring (Figure 1C-D, Figure 2B). Both the sensory and non-sensory cells of the lips are joined apico-laterally to each other by tight junctions (Figure 1H).

Sensory cells are flask-shaped and possess an oval central nucleus, scattered mitochondria, and few cisterns of rough endoplasmic reticulum (Figure 1G-H). The Golgi complex is composed of a stack of a few cisterns and associated vesicles. The cells bear many apical cilia, with a conventional 9+2 microtubular arrangement and a dense, short basal body with developed ciliary rootlets (Figure 1G). The basal plasmalemma of each sensory cell rests on the basal lamina, which forms a continuous fibrous layer supporting both sensory and other epithelial cells. Neurites were often observed in close proximity to the sensory cell membrane (Figure 1J-K). We never recognised axonal extensions of the basal plasmalemma of the sensory cells. Instead, the basal plasmalemma was always flat and approached by nerve fibres projecting from the brain. Occasionally, synapses could be identified (Figure 1J).

### Phylogenetic analysis of secondary sensory cells in chordates

#### Taxon sampling

The diverse patterns of secondary sensory cells and associated structures close to the mouth of tunicates presented a potentially large amount of phylogenetic information. To analyse this information we compiled a data matrix based on 19 characters covering 20 different species of chordates, of which 16 were tunicates. The cephalochordate *Branchiostoma floridae* (amphioxus), the two vertebrate agnathans *Lethenteron camtschaticum* (artic lamprey, synonym *Lampetra japonica*[34]) and *Eptatretus stoutii* (Pacific hagfish), and the vertebrate gnathostome *Danio rerio* (zebrafish) were selected as outgroups.

In amphioxus, two types of secondary sensory cells were described. Type II sensory cells are monociliated secondary sensory cells, are usually found individually or arranged in small clusters, and most may be mechanoreceptors [14,35]. Because these cells are scattered throughout the epidermis, we did not select them for our analysis. Instead, we considered the sensory cells that formed oral spines in larvae [15,17]. These sensory cells are monociliated and clustered so that 8–10 cilia together form a spine. Many spines are arranged in a discontinuous row on the outer margin of the mouth. Spines are able to respond to contact with debris by initiating the so-called cough response: the pharyngeal slits close as the pharynx contracts to expel water out of the mouth to dislodge the debris.

Among vertebrates, we considered two agnathans and a gnathostome in which hair cells of the lateral line has been extensively studied [36-39]. We selected this organ because it extends around the mouth region and possesses mechanosensory function.

In the context of tunicates, the number of species was too high with respect to the limited amount of characters and species belonging to the same genus possess the same type of secondary sensory cells; therefore we decided to consider only one representative species per genus (see Table 1 for the complete list of species). We chose six species of stolidobranch ascidians (*Botryllus schlosseri*, *Botrylloides leachi*, *Styela plicata*, *Polyandrocarpa zorritensis*, *Molgula socialis*, *Pyura stolonifera*), two species of aplousobranch ascidians (*Clavelina lepadiformis* and *Diplosoma listerianum*), and five species of phlebobranch ascidians (*Ciona intestinalis*, *Ascidiella aspersa*, *Phallusia mammillata*, *Chelyosoma productum*, and *Corella inflata*). The species *Oikopleura dioica* was chosen as the representative of the class Appendicularia and sensory cells of the circumoral ring were considered in the phylogenetic analysis. We did not consider the Langehrans cells because they are not located around or inside the mouth and they innervate their axon via electrical synapses (gap junctions) instead of classical chemical synapses [12]. Within the class of thaliaceans, the species *Pyrosoma atlanticum* and *Doliolum nationalis* were selected as representatives of the two orders Pyrosomatida and Doliolida; no species of the order Salpida was chosen because the only salp analysed, *Thalia democratica*, does not possess secondary sensory cells in the mouth [7]. Including *T*. *democratica* in the data matrix would result in a row consisting almost completely of characters coded “not applicable”.

**Table 1:** List of tunicate species considered in the cladistic analysis

**Traditionally recognized higher taxonomic groupings**				**Species**	**References**
Tunicata	Ascidiacea	Pleurogona	Stolidobranchiata	*Botryllus schlosseri*	[40]
				*Botrylloides leachi*, *B*. *violaceus*	[40,41]
				*Styela plicata*, *S*. *montereyensis*, *S*. *gibsii*	[3,42]
				*Polyandrocarpa zorritensis*	[3]
				*Molgula socialis*	[6]
				*Pyura stolonifera*, *P*. *haustor*	[3]
		Enterogona	Aplousobranchiata	*Clavelina lepadiformis*	[5]
				*Diplosoma listerianum*	[5]
			Phlebobranchiata	*Ciona intestinalis*	[4,5]
				*Ascidiella aspersa*	
				*Phallusia mammillata*	
				*Chelyosoma productum*	
				*Corella inflata*; *C*. *willmeriana*	
	Appendicularia			*Oikopleura dioica*; *O*. *albicans*	[43]
	Thaliacea	Pyrosomatida		*Pyrosoma atlanticum*	[7]
		Doliolida		*Doliolum nationalis*	[7]

#### Character coding

The formal coding of the characters is given in Tables 2 and 3; for all characters, 1 denotes presence and 0 denotes absence if not stated otherwise.

**Table 2:** Definition of characters used for the construction of the morphological character matrix

	**Character definition**
1	Single type of secondary sensory cells (present = 1, absent = 0)
2	Secondary sensory cells with a single cilium (monociliary) (present = 1, absent = 0)
3	Secondary sensory cells with two cilia (biciliary) (present = 1, absent = 0)
4	Secondary sensory cells with more than two cilia (multiciliary) (present = 1, absent = 0)
5	Cilia in multiciliary sensory cells of same length (0) / different lengths (1)
6	Microvilli or stereovilli on sensory cells (present = 1, absent = 0)
7	Microvilli on monociliary sensory cells (present = 1, absent = 0)
8	Stereovilli on monociliary sensory cells (present = 1, absent = 0)
9	Cilium of monociliary sensory cell surrounded by a ring of microvilli (0) or cilium eccentric to microvilli (1)
10	Microvilli on multiciliary sensory cells (present = 1, absent = 0)
11	Cilia in multiciliary sensory cells in a single line (0) or in multiple lines (1)
12	Accessory secretory cells in coronal organ (present = 1, absent = 0)
13	Supporting cells form a wall or crest alongside the coronal organ (present = 1, absent = 0)
14	Electron dense granules in sensory cells (present = 1, absent = 0)
15	Width of coronal organ uniform along oral rim (0) or wider at certain areas (1)
16	Accessory centriole in sensory cells (present = 1, absent = 0)
17	Tentacles or flaps present (present = 1, absent = 0)
18	Tentacles simple (0) / branched (1)
19	Secondary sensory cells in continuous row (present = 1, absent = 0)

**Table 3:** Morphological character matrix used for cladistic analysis

**Species**	**Character state**																		
	**1**	**2**	**3**	**4**	**5**	**6**	**7**	**8**	**9**	**10**	**11**	**12**	**13**	**14**	**15**	**16**	**17**	**18**	**19**
*Botryllus schlosseri*	0	1	0	0	-	1	1	0	1	-	-	0	1	0	0	1	1	0	1
*Botrylloides leachi*	0	1	0	0	-	1	1	0	1	-	-	0	1	0	0	1	1	0	1
*Styela plicata*	0	1	1	0	0	1	1	0	0	-	-	0	1	1	0	1	1	0	1
*Polyandrocarpa zorritensis*	0	1	1	0	0	1	1	0	0	-	-	0	1	1	0	1	1	0	1
*Molgula socialis*	0	1	1	0	0	1	1	0	0	-	-	0	1	1	1	1	1	1	1
*Pyura stolonifera*	0	1	1	0	0	1	1	0	0	-	-	0	1	1	1	1	1	1	1
*Clavelina lepadiformis*	1	0	0	1	0	1	-	-	-	1	0	0	1	0	0	0	1	0	1
*Diplosoma listerianum*	1	0	0	1	0	1	-	-	-	0	0	0	1	0	0	0	1	0	1
*Ciona intestinalis*	1	0	0	1	0	1	-	-	-	1	0	0	0	0	0	1	1	0	1
*Ascidiella aspersa*	1	0	0	1	0	1	-	-	-	1	0	1	0	0	0	0	1	0	1
*Phallusia mammillata*	1	0	0	1	0	0	-	-	-	-	0	0	0	1	0	0	1	0	1
*Chelyosoma productum*	1	0	0	1	0	1	-	-	-	1	0	1	0	0	0	0	1	0	1
*Corella inflata*	1	0	0	1	0	0	-	-	-	-	0	1	0	0	0	0	1	0	1
*Oikopleura dioica*	1	0	0	1	1	0/1	-	-	-	1	1	0	1	0	0	0	0	-	
*Pyrosoma atlanticum*	1	1	0	0	-	1	1	0	0	-	-	0	0	0	?	0	1	0	1
*Doliolum nationalis*	1	1	0	0	-	1	1	0	0	-	-	0	0	0	0	0	1	0	1
*Branchiostoma floridae*	1	1	0	0	-	1	0	0	0	-	-	0	0	0	-	1	1	0	0
*Lethenteron camtschaticum*	1	1	0	0	-	1	0	1	-	-	-	0	0	0	-	0	0	-	0
*Eptatretus stoutii*	1	1	0	0	-	1	1	0	-	-	-	0	0	0	-	?	0	-	0
*Danio rerio*	1	1	0	0	-	1	0	1	-	-	-	0	0	0	-	?	0	-	0

Matrix based on 19 characters derived from detailed ultrastructural studies of secondary sensory cells in different 20 species. For all the characters, “0” denotes absence and “1” presence (see Table 2 for character description); “-” was used when the character definition was not applicable to the species, “?” when the character state is not known. See Table 1 for references.

**Figure 3:**
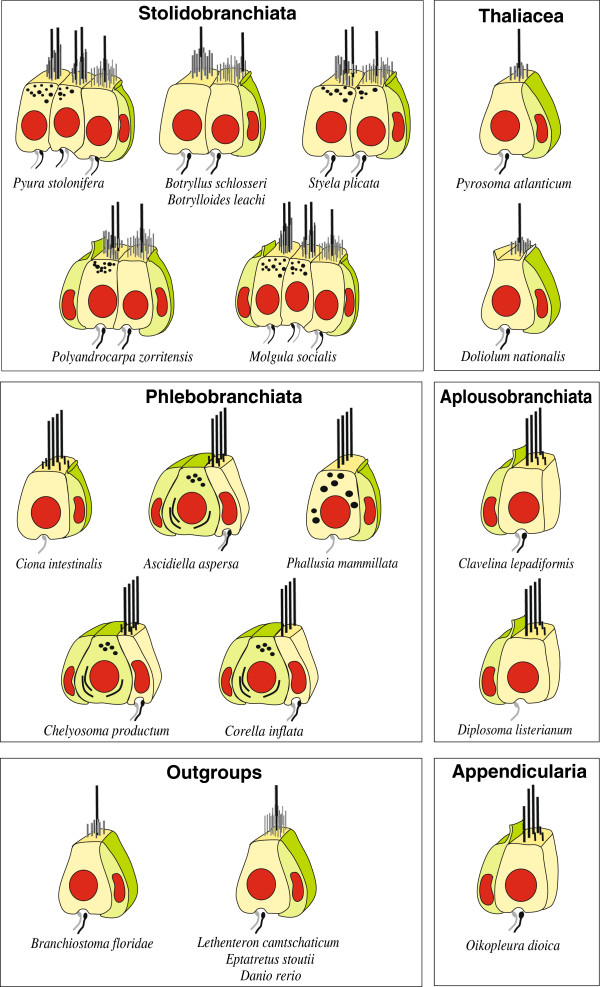
**Comparative schematic depictions of secondary sensory cells of representatives of all tunicate suborders and of outgroups.** Microvilli are drawn as shorter sticks compared to stereovilli, and cilia are represented by the longest sticks. Light green: supporting and secretory cells; light yellow: secondary sensory cells (modified from [3,5,7]).

The anatomical references used are listed in Table 1 for tunicates; for other species, references are cited in the previous section (Taxon sampling). Figure 3 presents schematic drawings summarising the main features of cells considered to be secondary sensory cells. Below, we describe each character highlighting key traits and variations in the sampled taxa.

1. *Single type of secondary sensory cells* (*present* = *1*, *absent* = *0*). The tunicate coronal organ is generally composed of a single type of secondary sensory cell, but stolidobranch ascidians may possess two or three different types of mechanoreceptors. For example, *Molgula socialis* exhibits a very complex condition. In this species, three different types of ciliated sensory cells have been identified: cells with a single cilium central to a group of short microvilli (type 1), and two types of cells bearing a more complex apical structure composed of two long cilia accompanied by a group of stereovilli graded in length. In these cells, stereovilli may form a crescent (type 2) or a complete ring around the two cilia (type 3). Cells follow a characteristic arrangement: types 2 and 3 are located towards the proximal side of tentacles and are mostly exposed to inflowing water, whereas type 1 is located more peripherally. Amphioxus oral spines and the lateral line organ of the three vertebrate species show a single type of sensory cells.

2. *Secondary sensory cells with a single cilium* (*monociliary*) (*present* = *1*, *absent* = *0*). All chordates considered except enterogon ascidians and *Oikopleura dioica*, possess sensory cells with a single cilium. It is noteworthy that in *Ciona intestinalis* (an enterogon ascidian species), differentiating coronal sensory cells reach the multiciliated state starting from a monociliated immature cell (unpublished data). This ontogenetic aspect of the transitory monociliated condition has not been considered in this phylogenetic analysis.

3. *Secondary sensory cells with two cilia* (*biciliary*) (*present* = *1*, *absent* = *0*). With the exception of *Botryllus schlosseri* and *Botrylloides leachi* (belonging to the same subfamily Botryllinae), stolidobranch species possess biciliated sensory cells. The pair of cilia is surrounded by a crescent or a ring of stereovilli that are graded in length.

4. *Secondary sensory cells with more than two cilia* (*multiciliary*) (*present* = *1*, *absent* = *0*). Enterogon ascidians and *Oikopleura* possess numerous cilia per cell. Cilia are not randomly distributed to form a bundle but constitute oriented rows parallel to coronal organ/circumoral ring arrangement.

5. *Cilia in multiciliary sensory cells of same length* (*0*) *or of different length* (*1*). Among species with multiciliated secondary sensory cells, *Oikopleura* displays the unique feature of cilia of different length in an orderly arrangement: cilia are shorter toward the cell edges and they are longer in the centre. This organisation confers a wavy aspect to the circumoral ring.

6. *Microvilli or stereovilli on sensory cells* (*present* = *1*, *absent* = *0*). With few exceptions (genera *Phallusia* and *Corella*), the apical surfaces of secondary sensory cells possess microvilli or stereovilli; these two apical specialisations are never simultaneously present in the same sensory cell. In some vertebrate species, the role of stereovilli in stimuli transduction has been identified [44,45]. Stereovilli are linked to each other and with the cilium (where present) by fibrillar links; this forms the structural device for transduction of the stimulatory force acting on the cilium to the site of mechanosensitive ion channels. In stolidobranch ascidians, extracellular radial filaments connecting the cilium or cilia to the surrounding stereovilli have been described [3,40] though the mechanism of signal transduction is not yet known. It is noteworthy that a loose fibrillar matrix is generally present also among microvilli and cilia.

7. *Microvilli on monociliary sensory cells* (*present* = *1*, *absent* = *0*). Generally, monociliated secondary sensory cells possess short, unbranched microvilli. Lamprey and zebrafish differ by possessing stereovilli instead.

8. *Stereovilli on monociliary sensory cells* (*present* = *1*, *absent* = *0*). Stereovilli associated to a single cilium have been described in hair cells of the lateral line organ in lamprey and zebrafish. This condition was not found in hagfish, amphioxus and tunicates. Stolidobranch ascidians are characterised by stereovilli in their coronal apical bundle but they associate with a couple of cilia.

9. *Cilium of monociliary sensory cell surrounded by a ring of microvilli* (*0*) *or cilium eccentric to microvilli* (*1*). Usually microvilli are short, of equal length, and form a corolla surrounding a central cilium. In *Botryllus schlosseri* and *Botrylloides leachi*, some coronal sensory cells possess an eccentric cilium that is laterally placed with respect to the group of microvilli.

10. *Microvilli on multiciliary sensory cells* (*present* = *1*, *absent* = *0*). The presence of microvilli is not constant in multiciliary sensory cells. Some enterogon ascidians (*Clavelina lepadiformis*, *Ciona intestinalis*, *Ascidiella aspersa*, *Chelyosoma productum*) show short, unbranched microvilli, accompanying a single row of cilia. By contrast, microvilli are absent in *Diplosoma listerianum*, *Phallusia mammillata* and *Corella inflata*. In *Oikopleura albicans*, a number of short microvilli accompany the cilia of sensory cells located on the ventral lip whereas those on the dorsal lip lack this specialisation.

11. *Cilia in multiciliary sensory cells in a single line* (*0*) *or in multiple lines* (*1*). In coronal sensory cells of enterogona ascidians, cilia of equal lengths are aligned to form a single line. Conversely, *Oikopleura dioica* presents a number of ciliary lines per cell and these cilia have different lengths that result in the wavy appearance of the sensory organ.

12. *Accessory secretory cells in coronal organ* (*present* = *1*, *absent* = *0*). Three enterogon ascidian species, *Ascidiella aspersa*, *Chelyosoma productum*, and *Corella inflata*, possess a more complex coronal organ that might be defined as “compound”. In these species the coronal organs consist of ciliated sensory cells flanked by secretory cells. The latter face towards the middle of the tentacles and do not form synapses with the nerve fibres that contact the ciliated sensory cells. Instead, these cells appear to be involved in protein synthesis, as evidenced by the presence of numerous, large cisterns of rough endoplasmic reticulum and Golgi complexes. The strict association of secretory cells with ciliated sensory cells suggests that the secretion mechanism might be activated by sensory cell stimulation, however the exact role of these cells has not been clarified by means of physiological studies.

13. *A wall or crest formed by supporting cells alongside the sensory organ* (*present* = *1*, *absent* = *0*). In some ascidians and in *Oikopleura dioica*, supporting cells possess an expanded apical lamina that delimits a sort of canal in which sensory cilia and stereovilli are found. In *Molgula socialis*, supporting cells are also polarised with respect to organ orientation: the inner supporting cells (facing the longitudinal axis of the tentacle, hence mostly exposed to inflowing water) possess an expanded apical lamina bordering the apical sensory structures whereas the outer supporting cells have shorter, irregular apical lamina. It has been hypothesised that the cytoplasmic laminae of supporting cells are functional devices to maximise the water flow against the hair bundles and facilitate the maintenance of laminar flows on them, optimising perception of particles entering the branchial basket [40].

14. *Electron dense granules in sensory cells* (*present* = *1*, *absent* = *0*). Some ascidian species exhibit sensory cells with cytoplasmic granules of different types (glycogen-like granules, multi-vesicular bodies and secretory granules). Their roles are not known.

15. *Width of sensory organ uniform along oral rim* (*0*) *or wider at certain areas* (*1*). *Molgula socialis* and *Pyura stolonifera* have a particularly complex coronal organ as it extends on branched tentacles and it is constituted by a variable number of rows of sensory cells. Generally, the basal part of a tentacle branch of the coronal organ exhibits fewer rows of sensory cells and the number of rows increases toward the tentacle branch apex.

16. *Accessory centriole in sensory cells* (*present* = *1*, *absent* = *0*). In many of the species considered, sensory cilia possess a basal body in which two centrioles are recognisable.

17. *Tentacles or flaps* (*present* = *1*, *absent* = *0*). All ascidians possess a crown of tentacles at the base of the oral siphon. In thaliaceans, *Pyrosoma atlanticum* has a dozen of flaps and a single ventral tentacle whereas *Doliolum nationalis* possesses only flaps. Amphioxus has velar tentacles as an adult. *Oikopleura dioica* does not possess tentacles or flaps but the mouth is delimited by two lips. Vertebrates do not exhibit tentacles or flaps.

18. *Tentacles simple* (*0*) / *branched* (*1*). Only the species *Molgula socialis* and *Pyura stolonifera* possess branched tentacles. If tentacles are present in other tunicates and in amphioxus, they are not branched and are usually cylindrical in cross section.

19. *Secondary sensory cells in continuous row* (*present* = *1*, *absent* = *0*). The coronal organ of tunicates is composed of sensory cells, which are adjacent to each other along the entire coronal organ. Conversely, oral spines in amphioxus and neuromasts in vertebrates are formed by focalised groups of sensory cells.

#### Phylogenetic analysis

**Figure 4:**
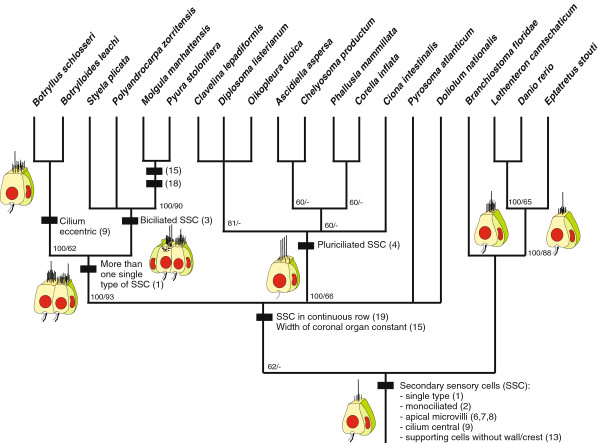
50%-majority-rule-consensus-tree of all most parsimonious trees obtained in PAUP* **version 4.0b10.** Major apomorphic changes are indicated along the branches by black rectangles with short descriptions of the character with corresponding character number in brackets (only numbers are given for *Molgula manhattensis* and *Pyura stolonifera*). Characteristic schematics correspond to those in Figure 3. Numbers indicate percentage of occurrences in the 50%-majority-rule-consensus-trees/jackknife percentages from 100 replicates with 30% character deletion.

We coded the 19 morphological characters listed above (Table 2) for the 16 tunicate genera (Table 1) and completed the matrix with scorings for the four outgroup species, which consisted of the cephalochordate *Branchiostoma floridae* and the vertebrates *Lethenteron camtschaticum*, *Eptatretus stoutii*, and *Danio rerio* (Table 3). The matrix has been deposited in Treebase (http://treebase.org/treebase-web) under the submission ID: 14197. 16 characters were parsimony informative, two characters were autapomorphic for *Oikopleura dioica* after we pruned a second species of the genus *Oikopleura* (*O*. *albicans*) from the taxa list, and one character was autapomorphic for *Diplosoma listerianum* (character 10 (*microvilli on multiciliary sensory cells*, see Table 3)). We removed *O*. *albicans* from the matrix because all character states except character 6 (*microvilli or stereovilli on sensory cells*, see Table 3) were identical in the two *Oikopleura* species, and we aimed for a more balanced ratio between taxa versus characters. The branch and bound analysis in PAUP* (4.0 b10) [33] resulted in 80 equally parsimonious trees with a tree length of TL=25, a consistency index of CI=0.76, and a rescaled consistency index of rCI=0.68. As a summary of the most parsimonious trees, the 50%-majority-rule-consensus-tree in Figure 4 shows a strongly supported monophyletic Stolidobranchiata, a strongly supported monophyletic Molgulidae plus Pyuridae, a strongly supported Vertebrata, a less strongly supported group consisting of Enterogona plus *Oikopleura*, and a weakly supported monophyletic Tunicata. Thus, despite the limits of restricting the phylogenetic analysis to characters linked to the oral secondary sensory cells and their supposed homologues, our phylogenetic analysis recovers major clades recognised within Tunicata and Chordata. This result demonstrates that the morphological variation observed in sensory organs based on secondary sensory cells contains phylogenetic information.

Several uncontroverted synapomorphies are reconstructed for the lineages of the monophyletic clades recovered within Tunicata (Figure 3). In the stem lineage of Enterogona plus *Oikopleura*, secondary mechanoreceptor cells became multiciliated (character # 4, Figure 3) whereas in the stem lineage of Stolidobranchiata, secondary mechanoreceptors cells diversified and more than a single type of such cells is present in the coronal organ (character # 1, Figure 3). This diversification of cell types is then accentuated again in the stem lineage of botryllid species where the position of the cilium in relation to the microvilli diversified (character # 9, Figure 3). In the stem lineage of the solitary styelids, molgulids, and pyurids, secondary mechanoreceptor cells became biciliated (character # 3, Figure 3). Thus, we infer a diversification of secondary mechanoreceptor cells in stolidobranch ascidians.

## Discussion

### The circumoral secondary sensory cells of appendicularians

Our morphological study suggests that, based on their corresponding positions, morphologies, and functions, appendicularian secondary sensory cells of the circumoral ring constitute the homologue of ascidian coronal organs. In the two analysed oikopleurid species, the circumoral ring is located around the oral aperture; in ascidians, the coronal organ has a similar position and is located around the mouth (oral siphon) aperture, in the ectoderm anterior to the pharynx [42,46]. Moreover, the embryonic primordia of both coronal organ and circumoral ring express orthologs of vertebrate placodal genes [22,24].

The circumoral ring cells are directly exposed to the inhaled water flow because they are not covered by the matrix that constitutes the external “house”. This is a sheath similar to a tunic in composition, in which appendicularians live and which serves as a concentration apparatus [43,47]. Water is pumped through the house by undulatory movements of the tail. Simultaneously, a ciliary current draws water enriched with food particles into the mouth. The food is trapped in mucus and ingested while the water passes out through two ventrolateral spiracles. This additional characteristic renders the circumoral cells comparable in position to that of the coronal organ. In ascidians and thaliaceans, the tunic layer envelopes the entire external animal surface including part or the inner wall of oral aperture, but the tunic ends just anterior of the position of the coronal organ so that coronal sensory cells are directly exposed to the water current [5,40,48]. This is different from what generally occurs in tunicate mechanoreceptor organs based on primary sensory cells, in which sensory elements are covered by either the outer tunic or by acellular cupulae [3,48].

Both circumoral cells and coronal cells are secondary receptors and synapses were clearly identified at the base of coronal sensory cells [46], though the evidence is less obvious in the case of the base of the circumoral ring cells (see Figure 1J). Nevertheless, axons extending directly from the base of circumoral ring cells were never found [8,43]. Finally, the circumoral ring and the coronal organ share similar mechanoreceptor functions linked to the alimentary activity: they are responsible for the expulsion of unwanted particles that incidentally enter the mouth with the water flow. In ascidians, this response is the typical squirting with muscle involvement. In appendicularians, no muscles reaction or squirting behaviour occurs, but the reflex consists in ciliary reversals that cause beating arrest, changing of the direction of the ciliary power stroke, water flow inversion, and expulsion of particles [10,43]. A peculiar feature of circumoral ring cells is the arrangement of their apical cilia. The multiple cilia are of different lengths in a cell with the shorter cilia being situated toward the cell border and the longer in the centre. This organisation confers a wavy aspect to the circumoral ring, has never been observed before in tunicates, and represents a novel form of organisation among the wide variability of apical structures in secondary sensory cells.

For all these reasons, we propose that the appendicularian oral sensory system is homologous to ascidian and thaliacean coronal organs.

### Phylogenetic information inferred by tunicate secondary sensory cells

While the traditional taxonomy of tunicate taxa is highly refined and the respective literature is substantial (e.g., [49]), phylogenetic analyses of exclusively morphological characters of tunicate taxa are rare and problematic. For example, Moreno and Rocha [50] presented a cladistic analysis of tunicate taxa on the level of traditionally recognised families. With the difficulties associated with coding for higher taxa and the principal reliance on characters traditionally used in this study, their resulting phylogeny was poorly resolved, with merely Stolidobranchiata and Aplousobranchiata of the traditionally recognised higher tunicate taxa being recovered as monophyletic. Another attempt at cladistically analysing morphological characters by Moreno and Rocha [50] predominantly focused on the taxon Aplousobranchiata. Our present phylogenetic analysis is not intended as a comprehensive cladistic analysis of tunicate taxa but as a preliminary test for phylogenetic information content in a recently discovered sensory system. The formal cladistic analysis of our data matrix is congruent with traditional taxonomy and other morphological analyses in supporting the monophyly of Stolidobranchiata [2,49,51-54]. This indicates that the characters coded do indeed contain a phylogenetic signal and could therefore be useful in future attempts to cladistically resolve tunicate phylogeny based on a broader character sampling. In comparison to some recent molecular phylogenies showing that the monophyly of Stolidobranchiata is about the only corner stone recovered in most molecular systematic studies, the position of Appendicularia is extremely uncertain and there is no unambiguous support for monophyly of Phlebobranchiata, Aplousobranchiata, Thaliacea, or Enterogona [52,55,56]. Thus our cladistic analysis, albeit limited to a single organ system, mirrors the general picture seen in many recent molecular studies and strongly indicates the presence of phylogenetic information in our data.

### Evolutionary implications

The phylogenetic hypothesis derived from the analysis of morphological characters pertaining to oral secondary sensory cells implies some interesting character transformations. Most notably there is a diversification of sensory cell types in the stem lineage of Stolidobranchiata from a single cell type as the plesiomorphic condition in the ground pattern of Tunicata (see Figure 3). This diversification coincides with a considerable elaboration of the branchial basket in the form of internal, longitudinal blood vessels and folds that improve the filtration efficiency by increasing the surface area of the branchial basket [57-60]. Within Thaliacea, salps have secondarily lost the coronal organ and, in contrast to other thaliaceans, no secondary sensory cells are found around the mouth opening. It is interesting that here again a drastic change of feeding mode coincides with this loss of the coronal organ: different from all remaining thaliaceans, salps feed by using their substantial body muscles instead of cilia to create the feeding current [61]. Coincident with this new mode of feeding is a reduction of the branchial basket to a single pair of large gill openings, a dorsal gill bar, and a sturdy mucus net [62]. In contrast, the evolutionary origin of multiciliarity in the secondary sensory cells in Enterogona and Appendicularia cannot be easily related to feeding biology because this group contains the Aplousobranchiata with a simple branchial basket as well as Phlebobranchiata with more complicated forms. Interestingly, Appendicularia show unique morphology of their secondary sensory cells, as these possess cilia of different lengths alongside apical microvilli. Appendicularia also have a unique mode of feeding that uses an external house to sort and concentrate particles before they enter the mouth [63-65]. If the distribution of character states revealed in our studies is mapped on recent molecular phylogenies, the inferred character transformations would be essentially the same as sketched here. An exception would be that multiciliarity in appendicularians and Enterogona would be interpreted as convergent whereas appendicularians are grouped with Stolidobranchia (e.g., [52,55]). In conclusion, it seems clear that the evolution of sensory cells in the coronal organs of tunicates is intimately correlated with the evolution of the respective feeding system. The precise nature of this correlation remains to be investigated in greater depth.

## Conclusions

We investigated the circumoral ring of secondary sensory mechanoreceptive cells in two appendicularian species in morphological detail. We discovered a unique arrangement of multiple apical cilia that results in a wavy appearance of the organ perpendicular to the incoming water flow. Based on the similarities in position, cellular composition, connections to the nervous system, and presumed function, we suggest that the circumoral ring of mechanoreceptors in appendicularians is homologous to the coronal organs of other tunicates. We analysed morphological characteristics of the tunicate coronal organs cladistically comparing them to oral spine cells of amphioxus and hair cells of vertebrates as outgroups. Despite being restricted to a single organ system, the support of traditionally and molecularly recognised clades, demonstrates the presence of phylogenetic signal in this data set. We show that the secondary sensory cells in tunicates diversify in the course of evolution from a simple monociliated cell and that this diversification is correlated to the evolution of different feeding strategies in different tunicate lineages.

## Competing interests

The authors declare that they have no competing interests.

## Authors’ contributions

All authors had full access to all the data in the study and take responsibility for the integrity of the data and the accuracy of the data analysis. The concept and design of the study was developed by TS and LM. FC and FR acquired the data. The analysis and interpretation of data was performed by LM, PB, and TS. The manuscript was drafted by LM and FG. PB and FG critically revised the manuscript for intellectual content. LM and TS obtained the funding for this work. Administrative, technical, and material support was provided by FC and FR. The study was supervised by LM. All authors read and approved the final manuscript.
